# Exploring the mechanisms of acupuncture in improving cognitive function in post-COVID-19 myalgic encephalomyelitis/chronic fatigue syndrome: study protocol for a randomized controlled trial using multimodal MRI

**DOI:** 10.3389/fneur.2026.1793397

**Published:** 2026-06-03

**Authors:** Tingting Luo, Yang Luo, Liang Huang, Hongjiao Jin, Yi An, Jinwei Huang, Kun Luo, Yan Guo, Dan Wang, Dafeng Liu, Xi Wu

**Affiliations:** 1 Acupuncture and Tuina College, Chengdu University of Traditional Chinese Medicine, Chengdu, China; 2 Department of Rehabilitation, The Thirteenth People’s Hospital of Chongqing, Chongqing, China; 3 Department of Pediatrics, Chongqing Health Center for Women and Children, Women and Children’s Hospital of Chongqing Medical University, Chongqing, China; 4 Department of Science and Teaching, Public Health Clinical Center of Chengdu, Chengdu, China

**Keywords:** acupuncture, cognitive function, functional connectivity, hippocampus, multimodal MRI, myalgic encephalomyelitis/chronic fatigue syndrome, neurochemical, post-COVID-19

## Abstract

**Background:**

Myalgic encephalomyelitis/chronic fatigue syndrome (ME/CFS) is a common sequela following COVID-19. Although cognitive dysfunction is one of the most debilitating symptoms in ME/CFS, effective therapies are limited. Acupuncture is an important complementary and alternative therapy for ME/CFS and has been shown to have positive effects on cognitive dysfunction in other diseases. However, the effect and mechanism of acupuncture in treating cognitive dysfunction in post-COVID-19 ME/CFS(PCME/CFS) remain unclear. In this study, we designed a randomized controlled trial to evaluate the efficacy of acupuncture treatment in improving cognitive function in PCME/CFS and to investigate the neural mechanisms of acupuncture using multimodal magnetic resonance imaging (MRI) techniques.

**Methods:**

A total of 129 patients and 30 healthy controls (HCs) will be enrolled. The 129 patients with PCME/CFS will be randomly assigned in a 1:1:1 ratio to a verum acupuncture (VA), sham acupuncture (SA), or a waitlist control group. Participants in the VA and SA groups will receive three sessions of treatment per week for 8 weeks, while patients in the waitlist control group will be treated after the 8-week waiting period. The primary outcome is the change in the Symbol Digit Modalities Test (SDMT) score from baseline to week 8. The secondary outcome measures include changes from baseline to endpoint (week 8) in cognitive performance as assessed by the Digit Span Test (DST), Trail Making Test (TMT), Rey Auditory Verbal Learning Test (RAVLT), Rey–Osterrieth complex figure test (RCFT), Stroop Color and Word Test (SCWT), phonemic fluency test, category fluency test, action fluency test, and 30-item Boston Naming Test (BNT-30). In addition, changes in hippocampal metabolites and resting-state functional connectivity(RSFC) will be examined using ^1^H-magnetic resonance spectroscopy(^1^H-MRS) and functional MRI (fMRI), respectively. Moreover, the Multidimensional Fatigue Inventory (MFI-20), Pittsburgh Sleep Quality Index (PSQI), Generalized Anxiety Disorder 7-item scale (GAD-7), 24-item Hamilton Depression Scale (HAMD-24), and 36-Item Short Form Survey (SF-36) will also be assessed at baseline and week 8.

**Discussion:**

The results of this study will provide preliminary evidence regarding the efficacy of acupuncture therapy in improving cognitive function in PCME/CFS and will explore whether acupuncture improves cognitive function in this disease by modulating metabolism and RSFC in the hippocampus.

**Clinical trial registration:**

www.clinicaltrials.gov, identifier: NCT07357688.

## Introduction

Myalgic encephalomyelitis/chronic fatigue syndrome (ME/CFS) is an illness characterized by profound fatigue lasting more than 6 months, post-exertional malaise (PEM), and unrefreshing sleep (1), affecting 0.68% of adults (2). ME/CFS usually leads to substantial functional decline, which is more pronounced during episodes of PEM (3, 4). Full recovery is rare among untreated patients with ME/CFS (5).

Viral infection is an important trigger for ME/CFS, including SARS-CoV-2 (6, 7). CFS-like symptoms are common chronic manifestations following COVID-19 (8). It was found that 86.7, 72.2, and 50.5% of COVID-19 survivors experienced fatigue, PEM, and cognitive problems, respectively, 6 months after infection (9). A systematic review of 52 studies reported that 45.2% of individuals with COVID-19 had CFS-like symptom clusters 1 month after illness onset (10). The retrospective analysis of clinical manifestations in patients experiencing persisting symptoms following COVID-19 showed that 43% of patients fulfilled the National Academy of Medicine (NAM) criteria for ME/CFS 8 months after COVID-19 (11).

ME/CFS is a multisystemic illness with diverse presentations (12), and cognitive dysfunction is one of the most debilitating symptoms, with 82 and 52% of ME/CFS patients experiencing concentration and memory problems, respectively (13). Objective cognitive assessment revealed deficits in psychomotor speed and visual and verbal memory among patients with ME/CFS (13–16). In addition, the impairment of information processing efficiency in ME/CFS appeared to be comparable to that observed in multiple sclerosis (MS) (17, 18). The investigation of the trajectory of cognitive symptoms following COVID-19 demonstrated that the symptom burden seemed to increase over time, persisting for at least 3 years after infection and manifesting as both worsening and new-onset cognitive problems (19). Cognitive symptoms—including memory and word-finding difficulties and reading problems—in patients who developed ME/CFS post-COVID-19 were reported to be highly severe for more than 20 months following infection (20). Consequently, there is a pressing need to address cognitive dysfunction in post-COVID-19 ME/CFS(PCME/CFS) patients.

The pathogenesis of ME/CFS is not fully understood at present, and a line of evidence indicates that the central nervous system is involved in the pathophysiology of ME/CFS (14, 21, 22). The hippocampus, as a part of the medial temporal lobe (MTL), plays a significant role in cognitive function, such as memory formation, executive function, and reward processing (23). Hippocampal abnormalities in ME/CFS have been documented in neuroimaging research. Structural magnetic resonance imaging (MRI) has revealed increased volumes in multiple hippocampal subfields, including the left subiculum head, presubiculum head, molecular layer hippocampus head, and whole hippocampal head, among individuals with ME/CFS; moreover, these changes have been associated with fatigue, cognitive problems, unrefreshing sleep, and pain (24–26). Functional neuroimaging research showed that ME/CFS patients had impaired resting-state functional connectivity (RSFC) in the right hippocampus, and the disruption was correlated with mental fatigue (27). While completing cognitive tasks, ME/CFS patients displayed greater activation and FC in the hippocampus, suggesting the compensatory role of this region (28, 29). In addition to structural and functional changes, abnormalities in hippocampal metabolites have also been observed in ME/CFS. Brooks et al. found decreased N-acetylaspartate (NAA) concentrations in the right hippocampus of patients with ME/CFS compared to healthy controls (HCs) using ^1^H-magnetic resonance spectroscopy (^1^H-MRS) (30). Another multi-voxel ^1^H-MRS study demonstrated that increased lactate (Lac) levels in the left hippocampus were associated with fatigue in individuals with ME/CFS (31). Similarly, elevated hippocampal subfield volumes (25), altered hippocampal microstructure, and reduced RSFC of the hippocampus (32) have been observed among patients with chronic fatigue following COVID-19, indicating that the hippocampus may be involved in the pathophysiology of post-COVID-19 fatigue.

Currently, there is no known cure for ME/CFS, and a symptomatic intervention is the main principle for the treatment of this syndrome. An array of strategies has been proposed for the management of ME/CFS, such as activity management, non-steroidal anti-inflammatory drugs (NSAIDs), antipsychotics, anticonvulsants, narcotics, and antidepressants (33, 34). However, these treatments primarily target non-cognitive symptoms such as fatigue, PEM, sleep disturbance, and pain, while their effects on cognitive problems are minimal (35). The efficacy of other approaches, such as immunoregulators, energy therapy, vitamin supplements, and dietary therapy for ME/CFS, is unclear due to limited and mixed findings (12, 36).

Acupuncture is an important complementary and alternative therapy for ME/CFS. The results of a meta-analysis showed that acupuncture could significantly improve fatigue, pain, depression symptoms, and quality of life in patients with ME/CFS compared to placebo controls, whether used alone or in combination with moxibustion or rehabilitation (37–39). However, research focused on the impact of acupuncture on cognitive function in ME/CFS is limited (38), despite evidence that acupuncture treatment can significantly improve cognitive dysfunction caused by neurological or neuropsychiatric disorders such as mild cognitive impairment (MCI), stroke, and insomnia (40–42). In addition, no one has evaluated the effect of acupuncture on cognitive function in PCME/CFS.

It has been documented that acupuncture therapy treats cognitive impairment by modulating hippocampal connectivity. Functional MRI (fMRI) studies have identified increased RSFC of the hippocampus among patients with Alzheimer’s disease (AD) after 10 min of acupuncture stimulation compared to pre-acupuncture measurements (43, 44). Wei et al. (45) also found that acupuncture treatment significantly enhanced hippocampal RSFC in AD patients, which was accompanied by improvements in general cognitive function. Similarly, Wang et al. reported that acupuncture treatment improved general cognitive function in patients with subjective cognitive decline (SCD) by increasing RSFC of the hippocampus (46). A modulatory effect on cerebral metabolites has also been observed for acupuncture. The findings of ^1^H-MRS research suggested that improvements in general cognitive function, visuospatial skills, and executive function following acupuncture treatment in patients with type 2 diabetes were associated with elevated levels of *γ*-aminobutyric acid (GABA) and NAA in the left basal ganglia, as well as reduced myo-inositol (mI) concentrations in the right basal ganglia (47). Guo et al. (48) revealed that acupuncture-induced cognitive improvements in individuals with chronic partial sleep deprivation-related cognitive dysfunction were correlated with increased levels of NAA and choline (Cho), as well as decreased GABA in the basal ganglia. Moreover, Zeng et al. (49) found that acupuncture treated cognitive dysfunction in patients with gynecological cancer by increasing NAA and Cho levels in the left hippocampus. However, whether acupuncture treatment can improve cognitive function in PCME/CFS patients by modulating hippocampal RSFC and metabolites is unclear.

Accordingly, this multimodal MRI trial is designed to assess the efficacy of acupuncture treatment in improving cognitive function in PCME/CFS patients and to explore whether such improvements occur by modulating hippocampal functional connectivity and metabolism.

## Methods and analysis

### Study design

**Figure 1:**
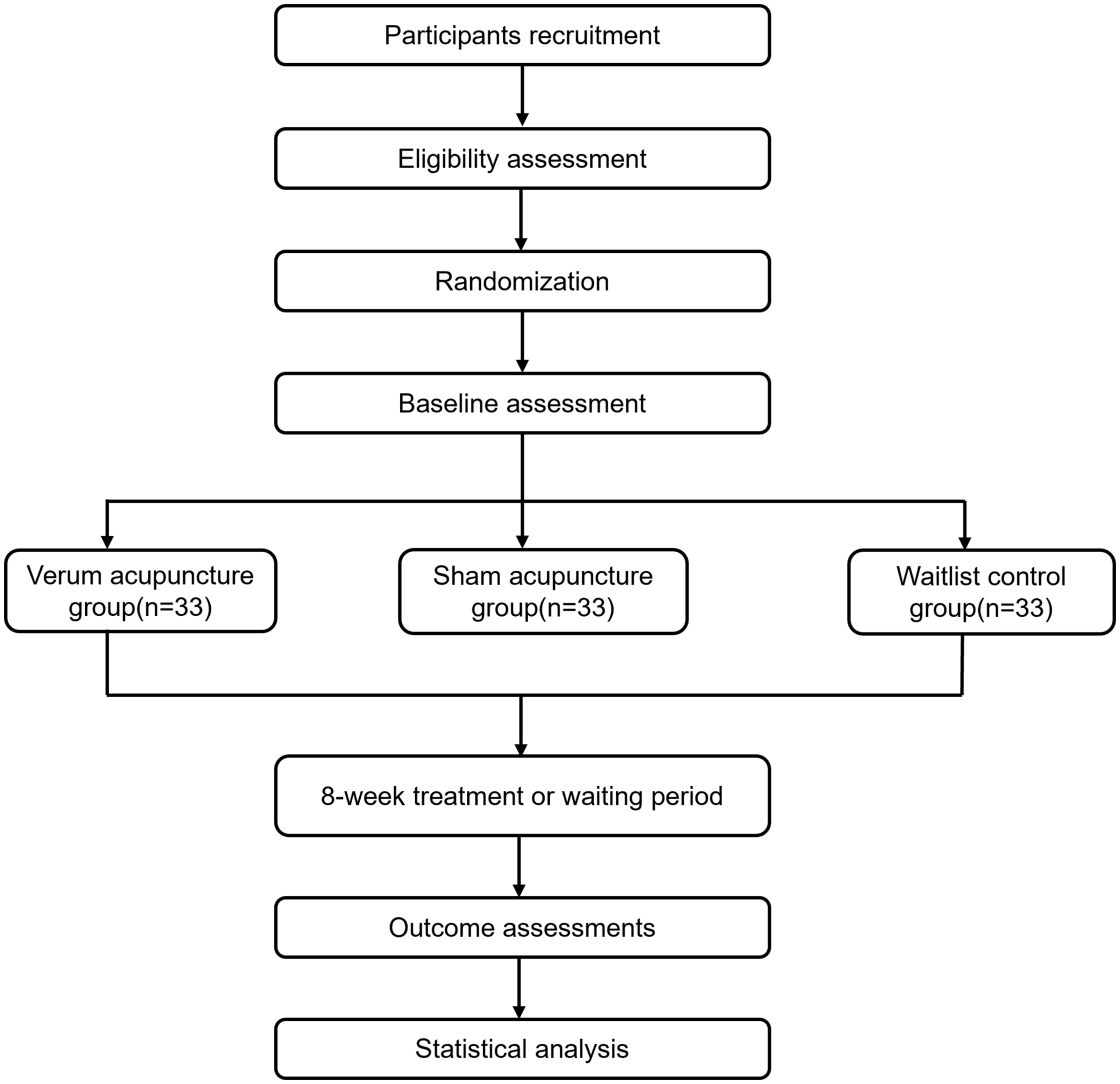
Flow chart of study procedure.

This is a prospective, three-armed randomized controlled trial using rs-fMRI and ^1^H-MRS, which will be jointly conducted by two hospitals in Chengdu. A total of 129 patients with ME/CFS occurring after COVID-19 and 30 HCs who have fully recovered from COVID-19 will be recruited. After baseline assessment, eligible patients will be randomly assigned to a verum acupuncture (VA), sham acupuncture (SA), or waitlist control group in a 1:1:1 ratio and will receive an 8-week intervention or wait. Figure 1 shows the flow chart of this trial. This study will be conducted in accordance with the Declaration of Helsinki, the Consolidated Standards of Reporting Trials (CONSORT) guidelines (50), and the Revised Standards for Reporting Interventions in Clinical Trials of Acupuncture (STRICTA) guidelines (51). This protocol has been reviewed and approved by the Ethics Committees of the Affiliated Hospital of Chengdu University of Traditional Chinese Medicine and the Public Health Clinical Center of Chengdu and has been registered at ClinicalTrials.gov (NCT07357688).

### Recruitment

Participants in this study will be prospectively enrolled from COVID-19 survivors who were hospitalized at the Public Health Clinical Center of Chengdu between 01 January 2020 and 31 December 2023, as well as from outpatients of the Affiliated Hospital of Chengdu University of Traditional Chinese Medicine and community residents in Chengdu. For hospitalized COVID-19 survivors, preliminary screening will be performed by two appointed investigators through a telephone interview. Then, a face-to-face interview and routine blood tests will be performed to identify eligible participants. For other recruitment sources, advertisements will be disseminated both online and offline via platforms such as WeChat, websites, and others. The investigators will screen eligible participants according to inclusion and exclusion criteria. The written informed consent form will be obtained from each participant prior to formal inclusion.

#### Participants

##### Diagnostic criteria

The diagnosis of ME/CFS will be made in accordance with the criteria developed by the NAM in 2015 (1), which include the following: (1) a substantial reduction or impairment in the ability to engage in pre-illness levels of occupational, educational, social, or personal activities that persists for more than 6 months and is accompanied by fatigue, which is often profound, of new or definite onset (not lifelong), not the result of ongoing excessive exertion, and not substantially alleviated by rest; (2) post-exertional malaise; (3) unrefreshing sleep; and at least one of either (4) cognitive impairment or (5) orthostatic intolerance.

##### Inclusion criteria for patients with PCME/CFS

Participants with PCME/CFS will be included if they meet all of the following criteria: (1) 18–60 years old; (2) fatigue that occurred during or after initial recovery from COVID-19, confirmed by nasopharyngeal swab RT-PCR or antigen testing, and lasting for at least 6 months; (3) fulfillment of the NAM criteria for ME/CFS; (4) being a native Chinese speaker; (5) right-handedness; and (6) willingness to participate in the study and provision of written informed consent.

##### Exclusion criteria for patients with PCME/CFS

Individuals meeting any of the following criteria will be excluded: (1) persistent fatigue occurring prior to COVID-19 infection; (2) a previous or current diagnosis of severe chronic diseases such as heart, kidney, or liver failure or tumors; (3) a previous or current diagnosis of neurological or psychiatric disorders such as neurodegenerative disease, stroke, epilepsy, bipolar disorder, or schizophrenia; (4) a previous or current diagnosis of endocrine or metabolic diseases such as hypothyroidism, adrenocortical hypofunction, or type 2 diabetes; (5) a previous or current diagnosis of autoimmune diseases such as systemic lupus erythematosus (SLE), Sjögren’s syndrome, or rheumatoid arthritis (RA); (6) chronic infection or inflammatory diseases such as acquired immunodeficiency syndrome (AIDS), chronic hepatitis, or irritable bowel syndrome; (7) substance or alcohol abuse; (8) obesity (BMI ≥ 28); (9) long-term use of immunosuppressants or glucocorticoids; (10) major surgery within the past year; (11) presence of metal or electronic implants; (12) claustrophobia; (13) pregnancy or lactation; (14) previous acupuncture treatment within the past 3 months; and (15) participation in another clinical trial within the past month.

##### Inclusion criteria for HCs

Participants meeting all of the following criteria will be included: (1) aged 18 to 60 years old; (2) full recovery from acute COVID-19, confirmed by nasopharyngeal swab RT-PCR or antigen testing; (3) right-handedness; (4) being a native Chinese speaker; and (5) willingness to participate in the study and provision of written informed consent.

##### Exclusion criteria for HCs

Individuals meeting any of the following criteria will be excluded: (1) persistent fatigue occurring prior to COVID-19 infection; (2) a previous or current diagnosis of severe chronic diseases such as heart, kidney, or liver failure or tumors; (3) a previous or current diagnosis of neurological or psychiatric disorders such as neurodegenerative disease, stroke, epilepsy, bipolar disorder, or schizophrenia; (4) a previous or current diagnosis of endocrine or metabolic diseases such as hypothyroidism, adrenocortical hypofunction, or type 2 diabetes; (5) a previous or current diagnosis of autoimmune diseases such as systemic lupus erythematosus, Sjögren’s syndrome, or rheumatoid arthritis; (6) chronic infection or inflammatory diseases such as AIDS, chronic hepatitis, or irritable bowel syndrome; (7) substance or alcohol abuse; (8) obesity (BMI ≥ 28); (9) long-term use of immunosuppressants or glucocorticoids; (10) major surgery within the past year; (11) presence of metal or electronic implants; (12) claustrophobia; (13) pregnancy or lactation; (14) previous acupuncture treatment within the past 3 months; and (15) participation in another clinical trial within the past month.

##### Withdraw criteria

Participants meeting any of the following criteria will be withdrawn from the study: (1) poor compliance, unwillingness to continue participation, or voluntary withdrawal; (2) occurrence of serious adverse events (SAEs) or complications that make continuation of the study impossible; and (3) failure to adhere to the treatment protocol or insufficient observation data that could affect data analysis.

#### Randomization and blinding

An appointed investigator who is not involved in participant recruitment, treatment, outcome assessment, or data analysis will generate the random sequence using SAS version 9.4 (SAS Institute, Inc) through a simple randomization method. The acupuncturist, who will not be involved in participant recruitment, will receive the grouping information for each participant from this investigator via short message service (SMS).

Participants in the VA and SA groups will be blinded to treatment allocation. VA and SA treatments will be performed in separate treatment rooms to reduce the risk of accidental unblinding. Participant recruitment, outcome assessment, imaging processing, and data collection, management, and analysis will be performed by independent investigators. The VA, SA, and waitlist control groups will be coded as A, B, and C, respectively, throughout the trial period until the completion of the primary analysis to ensure concealment of treatment assignment. It will be difficult to blind participants in the waitlist control group because they will not receive treatment immediately. A participant’s treatment allocation will be unblinded in the event of SAEs occurring during the trial.

The success of blinding will be evaluated for participants in the VA and SA groups at two time points (the first week and the 8th week after treatment) by asking them to guess their group allocation. The Bang’s blinding index (Bang’s BI) will be calculated according to the guessed proportion of each arm (52, 53).

#### Interventions

Healthy controls (HCs) will not receive any intervention during the trial period; clinical and brain imaging data will be collected only once. All interventions will be carried out in the outpatient department of the Affiliated Hospital of Chengdu University of Traditional Chinese Medicine. A trained and certified acupuncturist with at least 5 years of working experience will administer the interventions. The treatment protocol consists of 24 sessions, each lasting 30 min, administered over 8 weeks (three sessions per week). The acupuncturist will twirl and lift or thrust needles for 10 s every 15 min during each treatment session. The acupuncturist is not allowed to discuss the effects of acupuncture with the participants during the treatment period.

##### Verum acupuncture group (VA)

According to analyses of acupoint selection in acupuncture treatment for ME/CFS (54) and MCI (55), two sets of acupoints have been selected for the VA group. The first set includes Baihui (GV20), bilateral Shenmen (HT7), bilateral Neiguan (PC6), Qihai (CV6), Guanyuan (CV4), bilateral Zusanli (ST36), and bilateral Sanyinjiao (SP6). The second set consists of Sishenchong (EX-HN1), bilateral Ganshu (BL18), bilateral Pishu (BL20), bilateral Shenshu (BL23), and bilateral Taixi (KI3). One set of acupoints will be used in each treatment session, and the two sets of acupoints will be applied alternately. The locations of the acupoints conform to the 2021 National Standards of the People’s Republic of China (GB/T12346–2021) and are presented in Table 1.

**Table 1:** Locations and needling methods of acupoints used in the verum acupuncture group.

Acupoints	Locations	Needling methods
Baihui (GV20)	On the top of the head, 5 cun above the midpoint of the anterior hairline.	Subcutaneous insertion 0.5–0.8 cun; twirling the needle to elicit Deqi sensation.
Shenmen (HT7)	On the inner area of the wrist, at the ulnar end of the distal wrist crease, in the depression along the radial side of the flexor carpi ulnaris tendon.	Perpendicular insertion 0.3–0.5 cun; twirling or lifting and thrusting the needles to elicit Deqi sensation.
Neiguan (PC6)	On the inner area of the forearm, 2 cun above the distal wrist crease, between the palmaris tendon and the radial wrist flexor tendon.	Perpendicular insertion 0.5–1 cun; twirling or lifting and thrusting the needles to elicit Deqi sensation.
Qihai (CV6)	On the lower abdominal area, 1.5 cun below the navel, on the anterior midline.	Perpendicular insertion 1–1.5 cun; twirling or lifting and thrusting the needles to elicit Deqi sensation.
Guanyuan (CV4).	On the lower abdominal area, 3 cun below the navel, on the anterior midline.	Perpendicular insertion 1–1.5 cun; twirling or lifting and thrusting the needles to elicit Deqi sensation.
Zusanli (ST36)	On the outer side of the lower leg, 3 cun below the outer knee point and one finger-breadth lateral to the anterior border of the tibia.	Perpendicular insertion 1–2 cun; twirling or lifting and thrusting the needles to elicit Deqi sensation.
Sanyinjiao (SP6)	On the medial side of the shank, 3 cun above the tip of the medial malleolus, along the posterior medial edge of the tibia.	Perpendicular insertion 1–1.5 cun; twirling or lifting and thrusting the needles to elicit Deqi sensation.
Sishenchong (EX-HN1)	On the top of the head, 1 cun lateral to Baihui (GV20) in the anterior, posterior, left, and right directions; a total of 4 points.	Subcutaneous insertion 0.5–0.8 cun; twirling the needles to elicit Deqi sensation.
Ganshu (BL18)	On the back, below the spinous process of the 9th thoracic vertebra, 1.5 cun away from the posterior midline.	Oblique insertion 0.5–0.8 cun toward the spine; twirling the needles to elicit Deqi sensation.
Pishu (BL20)	On the back, below the spinous process of the 11th thoracic vertebra, 1.5 cun away from the posterior midline.	Oblique insertion 0.5–0.8 cun toward the spine; twirling the needles to elicit Deqi sensation.
Shenshu (BL23)	On the lower back, below the spinous process of the 2nd lumbar vertebra, 1.5 cun away from the posterior midline.	Perpendicular insertion 0.5–1 cun; twirling or lifting and thrusting the needles to elicit Deqi sensation.
Taixi (KI3)	The inner side of the ankle, in the depression between the tip of the medial malleolus and the tendo calcaneus.	Perpendicular insertion 0.5–1 cun; twirling or lifting and thrusting the needles to elicit Deqi sensation.

Participants will lie in a supine or prone position while receiving VA treatment. Disposable sterile acupuncture needles (0.30 mm × 40 mm or 0.30 mm × 0.25 mm, Hwato brand, Suzhou Medical Supplies Factory Co., LTD., Suzhou, China) will be used for the treatment. The needling method for each acupoint is shown in Table 1.

##### Sham acupuncture group (SA)

**Figure 2:**
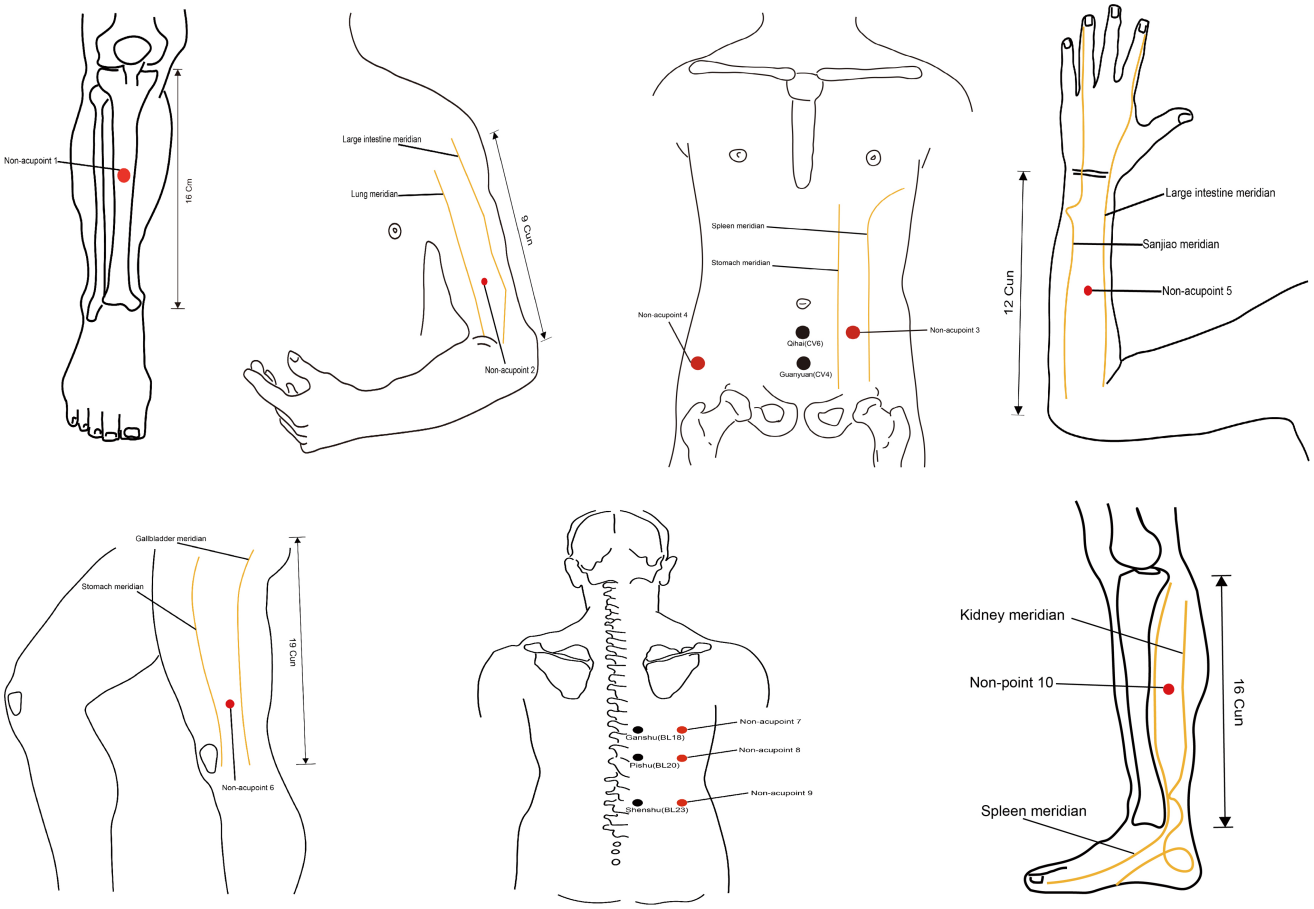
Locations of non-acupoints.

**Figure 3:**
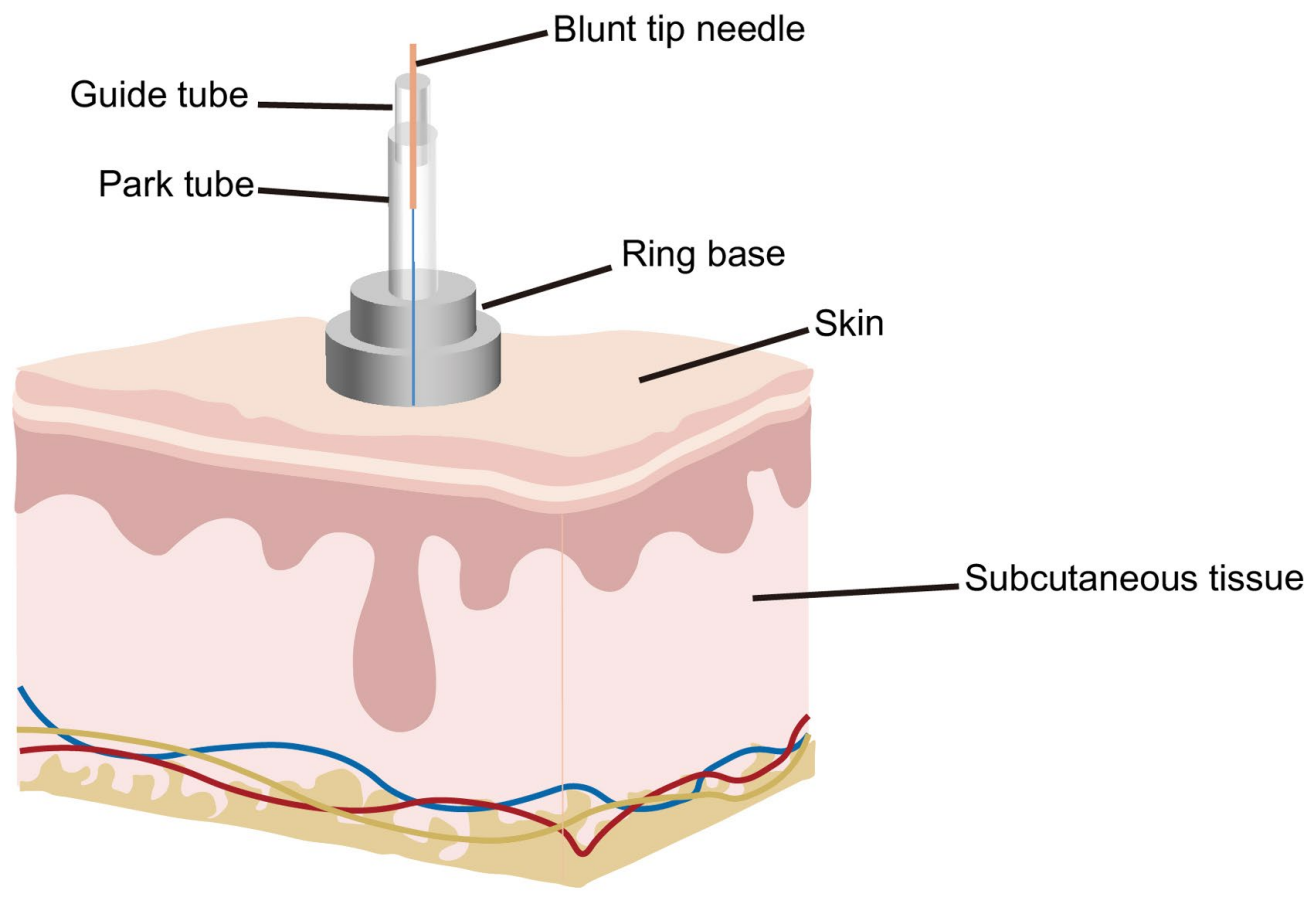
Park sham acupuncture device.

Non-penetrating acupuncture at non-acupoints will be performed using the Park sham acupuncture device (0.25 mm in diameter and 40 mm in length, Hwatuo, Suzhou, China) for participants in the SA group. Corresponding to the verum acupuncture group, two sets of non-acupoints will be applied alternately. The first set of sham acupoints includes bilateral non-acupoint 1, bilateral non-acupoint 2, non-acupoint 3, non-acupoint 4, bilateral non-acupoint 5, and bilateral non-acupoint 6. The second set consists of bilateral non-acupoint 7, bilateral non-acupoint 8, bilateral non-acupoint 9, and bilateral non-acupoint 10. The locations of the sham acupoints are shown in Table 2 and Figures 2, 3 illustrates the Park sham acupuncture device.

**Table 2:** Locations of non-acupoints used in the sham acupuncture group.

Non-acupoints	Locations
Non-acupoint 1	On the central area of the tibia, 6 cun above the tip of the lateral malleolus.
Non-acupoint 2	On the outer side of the upper arm, 4 cun above the elbow crease, at the midpoint between the large intestine meridian and lung meridian.
Non-acupoint 3	On the lower abdominal area, the left side of Qihai (CV6), at the midpoint between the stomach meridian and spleen meridian.
Non-acupoint 4	On the lower abdominal area, 6 cun lateral to Guanyuan (CV4) on the right side.
Non-acupoint 5	On the outer side of the forearm, 6 cun above the wrist crease, between the large intestine meridian and sanjiao meridian.
Non-acupoint 6	On the thigh area, 4 cun above the patellar base, at the midpoint between the stomach meridian and gallbladder meridian.
Non-acupoint 7	On the back, 3 cun lateral to Ganshu (BL18).
Non-acupoint 8	On the back, 3 cun lateral to Pishu(BL20).
Non-acupoint 9	On the back, 3 cun lateral to Shenshu(BL23)
Non-acupoint 10	On the inner side of the lower leg, 8 cun above the tip of the medial malleolus, at the midpoint between the kidney meridian and spleen meridian.

During treatment, participants will lie in a supine or prone position, and the Park device will be attached to the skin over the sham acupoints through the ring base. The blunt and retractable needle will be inserted into the plastic tube, and the needle will retract into the handle when the blunt tip contacts the skin. Thus, there will be no Deqi sensation.

##### Waitlist control group

Participants assigned to the waitlist control group will be instructed not to receive any therapy during the 8-week waiting period, but regular health follow-up through telephone will be conducted once a week by an appointed investigator. Furthermore, participants will be able to report their feelings to the investigator at any time. For those whose symptoms worsen during the waiting period, specialists at the Affiliated Hospital of Chengdu University of Traditional Chinese Medicine will be contacted immediately for symptomatic treatment, and intensive symptom monitoring will be implemented until symptoms return to their previous level. For participants whose exacerbated symptoms show no significant improvement or who develop new illnesses, withdrawal from the study will be recommended, and they will be advised to seek appropriate treatment. Participants who complete the 8-week waiting period will receive the same acupuncture treatment as the VA group for free.

#### Sample size calculation

The PASS software (version 21.0.3) was used to calculate the sample size. Based on two acupuncture trials that used the Symbol Digit Modalities Test (SDMT) as the primary outcome (56, 57), we estimated that the mean and standard deviation of the change in the SDMT score at week 8 after randomization would be 3.75 ± 4.95, 2.45 ± 5.21, and 1.30 ± 4.35 for the VA, SA, and waitlist control groups, respectively. With *α* = 0.05, *β* = 0.2, a power of 0.8, and a two-sided test for group differences, 36 participants are required in each group. Assuming a 20% dropout rate, 43 cases are needed per group, resulting in a total sample size of 129 participants.

#### Imaging data acquisition

MRI scans will be performed at the Huaxi MR Research Center, West China Hospital. An experienced radiologist will acquire brain images using a 3.0 T Prisma scanner (Siemens Healthcare, Erlangen, Germany) with a 64-channel head coil. All participants will undergo a T2-weighted fluid-attenuated inversion recovery (FLAIR) scan to exclude cerebral organic lesions (such as stroke and tumor) prior to formal experimental scans. After the exclusion of cerebral organic lesions in each participant, high-resolution T1-weighted imaging (T1WI), ^1^H-MRS, and rs-fMRI data will be acquired sequentially. During imaging data acquisition, participants will be instructed to relax and stay awake with closed eyes, and foaming pads will be used to reduce head motion.

T1-weighted images will be acquired using a 3D Magnetization Prepared Rapid Gradient Echo (3D-MPRAGE) sequence with the following parameters: repetition time (TR): 1500 ms, echo time (TE): 1.87 ms, field of view (FOV): 256 mm × 256 mm, and slice thickness: 0.8 mm. Single-voxel ^1^H-MRS data will be obtained using a point-resolved spectroscopy (PRESS) sequence with the following parameters: TR: 2000 ms, TE: 30 ms, FOV:240 mm × 240 mm, volume of interest (VOI): 40 mm × 15 mm × 20 mm, and flip angle: 90°. The bilateral hippocampi will be selected as the regions of interest (ROIs), and saturation bands will be applied around the ROIs to suppress confounding signals from the surrounding cerebrospinal fluid (CSF), skull, and blood vessels. Procedures such as shimming and water suppression prior to formal data acquisition will be automatically performed by the MRI scanner. Spectral data, both with and without water suppression, will be obtained. We will use the internal water signal as a reference for eddy current correction and absolute metabolite quantification. The scan parameters for the rs-fMRI images will be as follows: gradient-recalled echo echo-planar imaging (GRE-EPI) sequence, TR: 2000 ms, TE: 37 ms, slice thickness: 2 mm, and FOV: 208 mm × 208 mm.

#### Imaging data preprocessing

##### ^1^H-MRS data

Raw MRS data will be analyzed using the linear combination model (LCModel, version 6.3-1H, http://s-provencher.com/lcmodel.shtml). A total of two investigators will examine the data independently. Only spectra that meet the following criteria will be included in the subsequent analysis: Signal-to-noise ratio (SNR) ≥ 10, full width at half maximum (FWHM) ≤ 0.08 ppm, and Cramer–Rao lower bound (CRLB) < 15%. The metabolites to be measured will include choline (Cho), total creatine (Cr), glutamate (Glu), glutamine (Gln), lactate (Lac), myo-inositol (Ins), N-acetylaspartate (NAA), glycerophosphorylcholine (GPC), and glutathione (GSH). The final metabolite concentration will be expressed in mmol/kg wet weight.

To eliminate the partial volume effect of cerebrospinal fluid (CSF), we will use the cortical thickness procedure named Advanced Normalization Tools (ANTs) to segment T1-weighted images of each participant to obtain the whole-brain proportions of the gray matter, white matter, and CSF. The proportions within the voxel will then be determined by overlaying the voxel coordinates onto the segmented T1-weighted images. Finally, CSF-corrected metabolite concentrations will be calculated according to the following formula: C*_cor_* = C*_raw_* × [V*_total_* / (V*_total_*-V*_CSF_*)], where *C_cor_* is the corrected results, *C_raw_* is the uncorrected results of the whole voxel, *V_total_* represents the total volume of the voxel, and *V_CSF_* is the volume of CSF.

##### rs-fMRI data

The preprocessing of rs-fMRI data will be performed using the Statistical Parametric Mapping (SPM) 12 (Wellcome Trust Centre for Neuroimaging; http://www.fil.ion.ucl.ac.uk/spm) and the Data Processing and Analysis of Brain Imaging (DPABI) toolbox according to the following steps: (1) removal of the first 10 rs-fMRI images to allow for signal stabilization; (2) slice-timing correction; (3) realignment for head motion correction (excluding participants with head motion > 3 mm or head rotation > 3°);(4) normalization of functional images into Montreal Neurological Institute (MNI) space through the DARTEL alignment method and resampling to a voxel size of 3 × 3 × 3 mm^3^; (5) regression of nuisance covariates (including Friston-24 head motion parameters, global mean signals, white matter signals, and cerebrospinal fluid signals); (6) smoothing using a Gaussian kernel with a full width at half maximum (FWHM) of 6 mm; and (7) band-pass filtering (0.01–0.08 Hz) of the time series for each voxel to remove low-frequency drift and high-frequency noise.

The bilateral hippocampi will be selected as seed regions to perform seed-to-voxel RSFC analysis. These regions will be extracted from the Automated Anatomical Labeling (AAL) atlas. FC between each seed region and all other brain voxels (excluding the seed clusters) will be calculated using the DPABI toolbox. The mean time series of the left and right hippocampi will be extracted separately, and Pearson’s correlation coefficients will be calculated between the mean time series of each seed and the time series of all other voxels. The resulting correlation coefficients will then be *z*-normalized using Fisher’s *r*-to-*z* transformation. Finally, an RSFC map for each seed region will be generated for each participant.

#### Physical activity monitoring

Physical activity (PA) is a major factor that triggers the exacerbation of symptoms in patients with ME/CFS. To control the influence of variation in PA on study results, accelerometer-based PA monitoring will be performed. All participants will be instructed to wear an ActiGraph wGT3X-BT accelerometer (ActiGraph LLC, Pensacola, FL, USA) on the left wrist at all times throughout the 8-week trial period, except during sleep or water-based activities such as showering and swimming. Moreover, two appointed investigators will contact participants through WeChat (a social media platform in China) video calls between 8:30 a.m. and 9:00 a.m. each day to ensure adherence. The accelerometer-collected data will be exported every 2 weeks.

ActiLife version 6.13.4(ActiGraph Corporation) will be used to download and analyze the accelerometer data. The raw data will be converted into counts of movement using 60-s epoch lengths(ct/min). Non-wear time will be defined as zero accelerometer counts for any continuous 90 min. A day will be considered valid if the wear time is ≥ 10 h. Summary measures will be calculated using the average values across all valid days, including total PA time (min/day >100 ct/min), sedentary time (min/day for ≤100 ct/min), light PA time (min/day for 100–2019 ct/min), moderate PA time (min/day for 2020–5,998 ct/min), and vigorous PA time (min/day for ≥5,999 ct/min).

#### Outcomes

##### Primary outcome measure

The primary outcome is the change from baseline to week 8 in the SDMT score.

##### Secondary outcome measures

1. Cognitive measures include the following: Baseline-to-endpoint (week 8) changes in the Digit Span Forward Test (DST-F), the Trail Making Test Part A(TMT-A), immediate and delayed recall of the Rey Auditory Verbal Learning Test (RAVLT), copy trial, immediate and delayed recall of the Rey–Osterrieth complex figure test (RCFT),the Trail Making Test Part B(TMT-B), the Digit Span Backward Test (DST-B), the Stroop Color and Word Test (SCWT), the phonemic fluency test, the category fluency test, the action fluency test, and the 30-item Boston Naming Test (BNT-30).
2. Imaging measures include the following: Changes in NAA, Cho, Glu, Gln, Lac, Ins, GPC, GSH, and RSFC of each hippocampus at week 8.
3. Clinical symptoms and quality of life measures include the following: Baseline-to-endpoint (week 8) changes in the Multidimensional Fatigue Inventory (MFI-20), Pittsburgh Sleep Quality Index (PSQI), Generalized Anxiety Disorder 7-item scale (GAD-7), 24-item Hamilton Depression Scale (HAMD-24), and 36-Item Short Form Survey (SF-36).

##### Adverse events and safety

Any adverse events (AEs) or serious adverse events (SAEs) occurring during the intervention, whether related to the intervention or not, will be recorded in case report forms (CRFs) in detail (including the onset pattern, onset time, duration, severity, frequency, and treatment). Investigators will assess the relationship between AEs or SAEs and acupuncture treatment according to factors such as cause, timing, and clinical course. Common AEs related to acupuncture (including fainting, stuck needles, broken needles, bent needles, bleeding, edema, and other minor AEs) will be treated by the acupuncturist immediately. For SAEs, such as stroke recurrence or organ damage, researchers will immediately stop the treatment and send the patient to the emergency department. SAEs will be reported to the ethics committee within 24 h. The research team will bear the treatment costs for all AEs and SAEs.

#### Statistical analysis

Statistical analysis will be conducted by an appointed statistician blinded to group allocation using R software (version 4.2.1; R Foundation). Intergroup comparisons of RSFC will be performed using the DAPBI toolbox. Normally distributed continuous variables will be reported as mean±standard deviation, while non-normally distributed data will be reported as median [P25 P75]. A *p*-value of <0.05(two-tailed) will be set as the significance level for all analyses.

The intention-to-Treat (ITT) protocol will be used for the analysis of all outcomes. The ITT population will include all participants who are randomized, regardless of treatment adherence or protocol deviations. The missing value will be handled using multiple imputations.

First, intergroup comparisons of demographic data (such as age, sex, and educational level), illness duration, estimated premorbid intelligence function, and PA will be conducted to examine comparability across the three groups. Categorical data will be analyzed using the χ^2^ test. Normally distributed continuous variables will be tested using one-way analysis of variance (ANOVA), whereas the non-normally distributed data will be analyzed using the Kruskal–Wallis test.

Analysis of covariance (ANCOVA) will be applied to the primary outcome and other cognitive measures, with age, estimated premorbid intelligence ability, and PA as covariates. ANCOVA with age and PA as covariates will be used for clinical measures and brain metabolites. Repeated measures ANOVA will be used to compare differences in hippocampal functional connectivity among the three groups. Age, sex, and years of education will be included as covariates. *Post hoc* analysis will be performed for RSFC with interaction effects. Multiple comparisons for hippocampal functional connectivity will be corrected using cluster-level family-wise error (FWE) correction, with a significance threshold set at a p-value of < 0.05. The χ2 test will be used to compare the rate of AEs and SAEs between the groups. Finally, Spearman correlation analysis will be performed to examine the relationships between cognitive outcome measures and imaging indicators exhibiting intergroup differences.

## Discussion

ME/CFS is a common sequela of COVID-19, and cognitive dysfunction is a predominant symptom affecting patients’ quality of life. To the best of our knowledge, this is the first multimodal MRI trial focusing on the effects of acupuncture treatment on cognitive function and the mechanisms underlying improvements in cognitive performance in ME/CFS occurring following COVID-19.

In this trial, two sets of acupoints will be used alternately to overcome acupuncture tolerance (i.e., repeated or prolonged stimulation of an acupoint may lead to the attenuation or disappearance of the therapeutic effect), which is a common phenomenon in clinical practice. A recent scoping review on the relationship between acupuncture dose and efficacy showed that the linear increase in acupuncture efficacy disappears for several neurological disorders after a certain number of acupuncture treatment sessions (58). An important mechanism underlying this tolerance is neural adaptation, which refers to a phenomenon of decaying neuronal activities in response to repeated or prolonged stimulation (59). This adaptation has also been observed in acupuncture therapy. An fMRI study investigating the neural mechanism underlying the cumulative effects of acupuncture indicated that there is an augmentation of neural activity at the initial stage of acupuncture stimulation, but deactivation is observed as the number of acupuncture stimulation sessions increases (60). An alternating acupuncture strategy is able to overcome this adaptation, to some extent, and maintains the sensitivity and responsiveness of patients to acupuncture treatment.

Acupuncture is a complex intervention, for which clinical effects are composed of specific and non-specific acupuncture effects. The purpose of including a sham acupuncture control in the acupuncture trial is to investigate the specific acupuncture effect. The combination of sham acupuncture and blinding helps control non-specific acupuncture effects (i.e., placebo effect) (61). Multiple sham acupuncture techniques have been developed and can be roughly divided into categories of penetrating and non-penetrating sham methods (62). Non-penetrating acupuncture at non-acupoints, the method used in this study, is the optimal control among available sham acupuncture techniques in terms of placebo effectiveness as it not only excludes key factors contributing to specific acupuncture effects (e.g., acupoint specificity and needling sensation) but also effectively maintains participant blinding (63). A recent network meta-analysis of 45 randomized controlled trials on chronic pain showed that non-penetrating acupuncture at non-acupoints had the lowest placebo effect compared to manual acupuncture, shallow needling at acupoints, and non-acupoint acupuncture, with 64.8% of the surface under the cumulative ranking curve (SUCRA) (64). The Park sham device is one of the most frequently used non-penetrating sham acupuncture methods in clinical trials (65). Validation studies of Park sham acupuncture have shown excellent blinding effectiveness, especially in combination with non-acupoints (66–68). Furthermore, the blunt needle tip briefly contacts the skin during insertion in practice and does not consistently stimulate the skin during needle retention; thus, the possibility of persistent activation of sensory afferent fibers is low. This sham acupuncture method has been widely used in high-quality acupuncture trials and has successfully revealed the efficacy of acupuncture in the treatment of chronic spontaneous urticaria, Parkinson’s disease-related fatigue, irritable bowel syndrome, and herniated disk-related chronic sciatica (69–72). Therefore, non-penetrating acupuncture at non-acupoints using the Park sham device will be applied in this trial.

Objective neuropsychological tests are selected as outcomes in this study. These tests are chosen because impaired performance has been detected in patients with post-COVID-19 syndrome (73–79) and because of their widespread use in clinical practice. Although cognitive decline has been found in patients with post-COVID-19 condition (PCC), or long COVID, using cognitive screening tools such as the Montreal Cognitive Assessment (MoCA) and the Mini–Mental State Examination (MMSE) (80–82), such impairment appears to be less common than slowed information processing speed. Case–control studies on PCC consistently found that patients had longer reaction times than healthy people in cognitive tasks across low to high cognitive load conditions (73, 83–85). Moreover, a prospective study demonstrated that COVID-19-related decreases in the MoCA score return to normal levels 1 year after infection (86). In particular, impairment in information processing speed has been consistently reported by all neuropsychological reviews of ME/CFS (13–16). The SDMT is an important instrument for assessing information processing speed (87). It is a valid and reliable method used to detect cognitive deficits and to assess intervention efficacy for multiple sclerosis (MS), which is characterized by impaired information processing speed (88, 89). Similar cognitive slowing to that observed in MS has been identified in patients with PCC (90). Thus, this test is used as the primary outcome in this study.

This study will also explore the efficacy of acupuncture treatment on fatigue, sleep, anxiety, depression, and quality of life using the MFI-20, PSQI, GAD-7, HAMD, and SF-36. These outcomes are selected because of the common occurrence of these symptoms and functional decline in ME/CFS (91). These results may provide some directions for future research.

The hippocampus is an important structure in the medial temporal lobe, involved in visuospatial memory formation, working memory retention, attentional control, and emotion regulation. These functions are supported by interactions between the hippocampus and other brain regions (92). In the neuroimaging field, the measurement of functional connectivity between brain regions can reflect information exchange among them, and fMRI is a powerful tool for investigating cerebral functional connectivity and has been widely applied to understand the pathophysiology of various diseases (93). rs-fMRI research on healthy people has demonstrated that there are extensive functional connections between the hippocampus and the red nucleus, cingulate cortex, and dorsolateral prefrontal cortex (94–97). Alterations in hippocampal RSFC have been associated with impaired cognitive performance in ME/CFS (27). Acupuncture treatment is able to improve cognitive abilities by modulating hippocampal connectivity (45, 46).

Currently, ^1^H-MRS is the only imaging technique that is able to noninvasively examine cellular metabolism and can reveal underlying molecular mechanisms of disease by measuring the levels of specific metabolites (98). Several studies suggest that neuroinflammation, mitochondrial dysfunction, and oxidative stress are involved in the pathophysiology of ME/CFS (99–103). For example, Nakatomi et al. found glial activation (a cellular characteristic of neuroinflammation) across widespread brain regions in ME/CFS patients using 11C-(R)-PK11195 positron emission tomography (PET), including the cingulate cortex, hippocampus, amygdala, thalamus, and pons; furthermore, these changes were associated with cognitive impairment (104). Similarly, VanElzakker et al. identified increased neuroinflammation in the middle and anterior cingulate cortex, medial frontal gyrus, precentral gyrus, basal ganglia, thalamus, and corpus callosum in patients with post-COVID-19 syndrome relative to healthy controls (105). Muscle biopsy and genomic assays have found morphological changes (106), reduced enzymatic activity (107, 108), and mtDNA mutations (109) in the mitochondria in ME/CFS patients.^1^H-MRS studies have found abnormally elevated levels of Lac (a product of anaerobic metabolism) in the right insular, thalamus, cerebellum, and lateral ventricle (31, 110). Peripheral blood studies have also shown that the levels of oxidative stress markers such as isoprostanes (111), peroxides (112), and 2,3-diphosphoglycerate (113) are significantly elevated in patients with ME/CFS, while hypoxanthine (an antioxidant) (114) is significantly reduced compared to healthy individuals. Moreover, ^1^H-MRS research has revealed decreased concentrations of GSH in the anterior cingulate cortex in ME/CFS, suggesting cerebral oxidative stress in ME/CFS (115). Consequently, the levels of NAA, mI, Cho, GPC (reflecting neuroinflammation), Lac (mitochondrial function), and GSH (oxidative stress) in the hippocampus will be measured in this trial.

Acupuncture may improve symptoms in PCME/CFS by modulating neuroinflammation, mitochondrial dysfunction, and oxidative stress in the central nervous system (CNS). A meta-analysis of 23 studies indicated that acupuncture treatment reduced neuroinflammation in the hippocampus in animal models of Alzheimer’s disease (AD) by modulating the expression of inflammatory cytokines, which contributed to improvements in cognitive behavior (116). Positive effects on oxidative stress have also been observed for acupuncture. Animal research showed that acupuncture significantly reduced the level of malondialdehyde and increased the level of superoxide dismutase, glutathione peroxidase, and catalase compared to sham acupuncture (117). Similarly, a recent acupuncture trial in fibromyalgia (FM), a condition with significant overlap in symptoms and pathophysiological mechanisms with ME/CFS, found greater increases in plasma intracellular oxidized GSH(GSSG), reduced GSH, and total GSH levels in the real acupuncture group compared to the sham acupuncture group (118). Furthermore, acupuncture could reverse hippocampal mitochondrial dysfunction in vascular dementia rats through modulating mitochondrial respiratory chain complex enzyme (complex I, II, IV) activities and cytochrome oxidase IV expression (119).

In conclusion, the results of this study can not only reveal the efficacy of acupuncture treatment in improving cognitive function in PCME/CFS but also provide insight into the underlying neural mechanism by which acupuncture therapy may enhance cognitive function in this disease. Ultimately, the findings may provide a new treatment option for PCME/CFS patients with cognitive dysfunction.
